# Evaluation of physicomechanical and optical properties of bioactive glass (BAG)-filled resin composites: an *in vitro* study

**DOI:** 10.2340/biid.v13.46233

**Published:** 2026-06-15

**Authors:** Yan Victor Silva de Santana, Emanuel Ewerton Mendonça Vasconcelos, Gabriela Queiroz de Melo Monteiro, Agna Roberta Xavier Bezerra, Luís Felipe Espíndola-Castro

**Affiliations:** a Dental School, Federal University of Pernambuco, Recife-PE, Brazil; b Faculty of Dentistry, Postgraduate Program in Dentistry, University of Pernambuco, Recife-PE, Brazil

**Keywords:** Resin composite, nanotechnology, solubility, hardness, color

## Abstract

**Objective:**

To evaluate the behavior of bioactive glass (BAG)-filled resin composites (Beautifil II / Shofu—BR and Beautifil Flow Plus / Shofu—BF) compared with conventional resin composites (Filtek Z350 XT / Solventum—ZR and Filtek Supreme Flowable Restorative / Solventum—FS) by means of microhardness, sorption, solubility, and color stability tests.

**Materials and methods:**

For these analyses, ten specimens were fabricated per group using a split metal mold (15 mm in diameter × 1 mm thickness). Microhardness (*n* = 10) was measured using a digital microhardness tester, based on the mean of three indentations per specimen under a 2.94 N load for 15 s. For the sorption and solubility tests, five out of the 10 specimens used in the microhardness assays were randomly selected (*n* = 5), and ISO 4049/2019 recommendations were followed. Color stability was assessed using a digital spectrophotometer by comparing specimens immersed in coffee (using the five specimens not selected for the sorption and solubility tests / *n* = 5) and distilled water (control, using the same specimens from the sorption and solubility tests), with measurements obtained at baseline, after 1 day, and after 1 week of immersion. Color change (ΔE) was calculated using CIELAB parameters.

**Results:**

For microhardness, the high-viscosity resin composites (ZR and BR) showed significantly higher mean values than their low-viscosity versions (*p* < 0.001). Although sorption differed significantly among groups (*p* = 0.001), all resin composites exhibited values within the limits established by ISO 4049/2019. No statistically significant differences were observed for solubility. Regarding color stability, after 7 days in coffee, BR and ZR exhibited the greatest color changes (ΔE). Despite the observed statistical differences, the changes remained within acceptable clinical limits.

**Conclusion:**

BAG-filled resin composites exhibit properties comparable to those of conventional resin composites. The ZR resin composite showed higher microhardness; however, for the other evaluated parameters (sorption, solubility, and color stability in coffee), the tested resin composites exhibited values within the limits recommended by ISO 4049:2019 and were comparable to those of the reference conventional high- or low-viscosity resin composite.

## Introduction

Resin composites are widely used for direct restorations in dentistry, enabling additive restorative techniques and minimally invasive procedures [1]. However, broader clinical use and advances in scientific knowledge have improved the understanding of the intrinsic limitations of resin composites, such as polymerization shrinkage, potential cytotoxicity, and marginal gap formation [2]. These limitations may contribute to clinically observed failures, including marginal degradation and the development of secondary caries [3]. Despite continuous material development, these limitations remain clinically relevant and continue to compromise the longevity and predictability of restorations, highlighting the need for alternative strategies to overcome such shortcomings [3]. In this context, “bioactive” resin composites have recently been introduced to the market with the aim of improving biocompatibility and dental remineralization, and reducing secondary caries rates [4]. Although these resin composites show promising potential, their clinical indications remain limited due to the lack of robust long-term evidence comparing their performance with conventional resin composites [5]. Only a limited number of studies have evaluated the physicomechanical properties of these materials [6–8].

“Bioactive” restorative resin composites are materials that incorporate additives with specific properties. Each formulation is tailored to optimize resin composite performance while preserving its primary function and integrating a bioactive component [4]. Bioactive resin composites are promising for their ability to deliver bioactive agents that release critical ions, helping restore the chemical imbalance that leads to dental mineral loss [5]. From a restorative perspective, bioactivity is typically achieved by incorporating ion-releasing components, such as calcium, phosphate, and fluoride, into the resin matrix. These ions can saturate the tooth–restoration interface, buffer acidic challenges, promote remineralization, and exert antibacterial effects [5]. Fluoride ions inhibit demineralization by facilitating the conversion of hydroxyapatite into fluorapatite, which is more resistant to acid attack, and by interfering with bacterial glycosyltransferase activity, thereby reducing biofilm virulence [5]. However, incorporating these components into the resin composite formulation can influence its physical, mechanical, and optical behavior in ways that are not yet fully understood.

Several compounds, such as silver nanoparticles (AgNPs) and bioactive glasses (BAG), have been investigated for their ability to confer bioactivity and antimicrobial properties to resin composites. However, there is concern that inadequate incorporation of these additives into the resin matrix may compromise the desired mechanical behavior [9]. In particular, the balance between bioactivity and mechanical integrity remains a critical challenge, as improvements in biological performance may occur at the expense of strength, hardness, or durability [9]. This concern is amplified by the complex oral environment, which exposes materials to diverse fluids and substances, potentially adversely affecting their physicochemical properties and color stability and, consequently, reducing their durability [10].

This interaction with the oral environment also influences staining of resin composite restorations, as color change depends on the type of substance with which the resin composite comes into contact [11]. Color stability and wear resistance are critical factors that not only affect restoration longevity, but also influence the clinical decision to replace them. Despite their importance, there is still limited evidence on how bioactive modifications impact these properties over time. In addition, shade matching, anatomic form, and abrasion resistance are parameters widely used to assess restoration quality, following the standards established by the United States Public Health Service (USPHS) [12].

However, it is important to note that not all evaluated criteria have the same clinical relevance in decision-making. While some parameters, such as marginal integrity, secondary caries, and fracture, are more directly associated with restoration failure and may indicate the need for replacement [3], others, such as color mismatch or minor surface wear, may still be considered clinically acceptable and manageable through repair or repolishing procedures [3, 13]. Therefore, the clinical interpretation of these criteria should consider not only the presence of alterations, but also their severity and impact on restoration longevity

The incorporation of bioactive technologies influences water interaction in resin composites, affecting sorption and solubility in the oral environment [14, 15]. In particular, the presence of ion-releasing fillers may increase water diffusion within the resin composite, as water uptake is often necessary to enable ionic exchange and bioactive functionality [8]. Although water uptake is associated with potential drawbacks, such as color change, hydrolytic degradation, reduced wear resistance, and deterioration at the resin–filler interface, it is not necessarily the primary factor determining clinical failure.

It is also important to recognize that resin composites are not completely impermeable, and a certain degree of water sorption is expected [14]. In some cases, water uptake may even partially compensate for polymerization shrinkage by promoting hygroscopic expansion, thereby reducing shrinkage stresses [16]. However, excessive or uncontrolled water sorption may negatively impact the resin composite by increasing plasticization, facilitating chemical degradation, and compromising long-term stability [16].

Accordingly, the aim of the present study was to compare the behavior of BAG-filled resin composites with conventional resin composites by means of microhardness, sorption, solubility, and color stability tests. The null hypotheses were that there would be no differences in (I) microhardness, (II) sorption, (III) solubility, or (IV) color stability among the tested resin composites.

## Materials and methods

BAG-filled resin composites, Beautifil Flow Plus (Shofu) and Beautifil II (Shofu), were evaluated and compared with the conventional resin composites Filtek Z350 XT (Solventum) and Filtek Supreme Flowable Restorative (Solventum) (Table 1).

**Table 1:** Composition of the resin composites investigated in the study.

Group	Resin composite	Composition	Classification	Lot
BF	Beautifil Flow Plus (Shofu, Kyoto, Japan)	Organic matrix: Bis-GMA, TEGDMA Inorganic matrix: S-PRG, fluoroaluminosilicate glass: 67.8 wt% and 47 vol%.	Low-viscosity, bioactive.	PN2001
BR	Beautifil II (Shofu, Kyoto, Japan)	Organic matrix: Bis-GMA, TEGDMA Inorganic matrix: S-PRG, fluoroaluminosilicate glass: 83.3 wt% and 68.6 vol%.	High-viscosity, Nanohybrid, bioactive.	PN1401
ZR	Filtek Z350 XT (Solventum, Minnesota, USA)	Organic matrix: Bis-GMA, UDMA, TEGDMA, Bis-EMA Inorganic matrix: Silica and zirconia: 78.5 wt% and 63.3 vol%.	High-viscosity, Nanoparticle universal.	2500200488
FS	Filtek Supreme Flowable Restorative (Solventum, Minnesota, USA)	Organic matrix: Bis-GMA, UDMA, TEGDMA, Bis-EMA Inorganic matrix: Silica and zirconia: 65 wt% and 46 vol%.	Highly esthetic, low-viscosity, nanoparticle.	251010044

S-PRG: Surface pre-reacted glass-ionomer; UDMA: urethane dimethacrylate; Bis-GMA: bisphenol A-glycidyl methacrylate; BisEMA: ethoxylated bisphenol A dimethacrylate; TEGDMA: triethylene glycol dimethacrylate.

### Sample size calculation and specimen preparation

For the sample size calculation, a previous study that also evaluated the microhardness of resin composites using protocols similar to those adopted in the present study was considered [17]. To detect statistically significant differences in microhardness among resin composites, a minimum detectable difference of 3.52 HV was established. Based on a mean standard deviation of ±2.22, a significance level of 5%, and a statistical power of 80%, a minimum sample size of 10 specimens per group was estimated. The sample size calculation was performed considering a test for comparison of more than two independent group means Analysis of Variance (ANOVA), using the tool available at: http://estatistica.bauru.usp.br/calculoamostral/calculos.php (University of São Paulo, Bauru, São Paulo, Brazil).

For the sorption and solubility tests, a sample size of five specimens per group was adopted, as recommended by ISO 4049:2019. For the color stability tests, the present study followed a methodology described in previous studies [14, 17–20]. Thus, from the ten specimens prepared for the microhardness test, five were randomly selected (using the platform https://www.sealedenvelope.com) for the sorption and solubility tests and subsequently immersed in water, serving as the control group. The remaining five specimens, not selected for these tests, were immersed in coffee for the evaluation of color stability.

Specimens were prepared using a split metal mold (15 mm in diameter × 1 mm thickness) positioned between two polyester matrix strips and two glass plates (500 g). Specimen dimensions followed ISO 4049:2019 recommendations.

A light compressive load was applied for 20 s to promote better adaptation of the resin composite within the mold and to standardize specimen surfaces (placement of a 500 g glass plate). Light curing was initially performed through a glass plate placed over a polyester matrix strip using a light-emitting diode unit (Radii-Cal CX, SDI, Victoria, Australia) for 40 s at an irradiance of 1,200 mW/cm² to promote initial curing. After this initial curing step, the glass plate was removed, and additional light curing was carried out directly on both top and bottom surfaces of the specimens for 40 s each, following the recommendations of ISO 4049:2019. Overlapping light exposures were performed to ensure complete coverage of the entire specimen surface. After removal from the metal mold, excess material was removed using silicon carbide abrasive papers in ascending grit sequence (#600, 1,000, and 1,500). Final specimen dimensions were confirmed with a digital caliper (±0.01 mm; MDC-25 M, Mitutoyo, Tokyo, Japan).

Specimens were ultrasonically cleaned (Cristófoli, Paraná, Brazil) in distilled water for 10 min and gently dried with filter paper.

### Vickers microhardness analysis

Vickers microhardness (VHN) was measured using a digital microhardness tester (ISH-MR 150/INSIZE, São Paulo, Brazil). The indenter was positioned at the center of each specimen (*n* = 10), and a load of 2.94 N was applied for 15 s [14, 17–20]. Three indentations were performed per specimen, with 5 mm between indentations. The mean of the three measurements was calculated and used as the specimen microhardness value [10].

### Sorption and solubility assessment

After the microhardness evaluation, five specimens (*n* = 5) were randomly selected for the sorption and solubility tests. Specimens were placed in a desiccator containing silica gel at 37 ± 2°C, according to ISO 4049:2019. After 24 h, specimens were maintained at 23 ± 2°C for an additional 2 h. All specimens were weighed on an analytical balance (±0.01 mg; AUW 220D, Shimadzu Analytical Balance, Tokyo, Japan) repeatedly until a constant mass was obtained (m1), defined as a variation of < 0.1 mg. This constant mass (m1) was considered the initial specimen mass and was expressed in micrograms (µg).

The diameter and thickness of each specimen were measured with a digital caliper (±0.01 mm; MDC-25 M, Mitutoyo, Tokyo, Japan) at four points. Specimen volume (V) was calculated using the mean diameter and thickness values. Specimens were then stored in 50-mL Falcon tubes containing distilled water and maintained at 37°C for 7 days. Subsequently, specimens were removed, gently dried with filter paper, weighed again, and the mass recorded (m2). Specimens were returned to the desiccator and weighed repeatedly (daily) until a constant mass (m3) was reached. Sorption (SP) and solubility (SL) (µg/mm³) were calculated using the following equations:


$$\mathrm{SP}=(m_{2}-m_{1})/V\text{ and }\mathrm{SL}=(m_{1}-m_{3})/V$$

where the specimen volume was calculated in mm³ (*V* = π*r*²h), in which *r* is the mean specimen radius (diameter/2) and h is the mean specimen thickness.

### Color stability

The color stability evaluation was based on methodologies described in previous studies [14, 17–20]. Color assessment was performed by a single operator at three different time points: (1) before immersion, (2) after 1 day, and (3) after 1 week of storage in distilled water and coffee. The five specimens (*n* = 5) immersed in distilled water for the sorption and solubility tests served as the control group. The remaining five specimens, which had undergone the Vickers microhardness test, were immersed in coffee. Coffee was prepared by dissolving 0.51 g of instant coffee powder in 50 mL of distilled water (Nescafé, Nestlé, São Paulo, SP, Brazil).

The CIELAB system comprises three axes: L* (lightness, from 0 = black to 100 = white), a* (from −a = green to +a = red), and b* (from −b = blue to +b = yellow). Color differences were evaluated using both the CIELAB (ΔE) and CIEDE2000 (ΔE₀₀) color difference formulas:


$$\Delta \text{E}=\sqrt{{\text{L}}^{2}+{\text{a}}^{2}+{\text{b}}^{2}}$$

*And*


$$\Delta {\text{E}}_{00}=\sqrt{{\frac{\text{L’}}{{\text{K}}_{\text{L}}{\text{S}}_{\text{L}}}}^{2}+{\frac{\text{C’}}{{\text{K}}_{\text{C}}{\text{S}}_{\text{C}}}}^{2}+{\frac{\text{H’}}{{\text{K}}_{\text{H}}{\text{S}}_{\text{H}}}}^{2}+{\text{R}}_{\text{T}}\frac{\text{C’}}{{\text{K}}_{\text{C}}{\text{S}}_{\text{C}}}+\frac{\text{H’}}{{\text{K}}_{\text{H}}{\text{S}}_{\text{H}}}}$$

where

∆L = differences in lightness

∆a = differences in red–green coordinate

∆b = differences in the yellow–blue coordinate

ΔC′: difference in chroma (color saturation).

ΔH′: difference in hue (color tone).

S_L: weighting function for lightness differences.

S_C: weighting function for chroma differences.

S_H: weighting function for hue differences.

k_L, k_C, k_H: parametric factors that adjust the influence of lightness, chroma, and hue, respectively (typically set to 1 under standard conditions).

R_T: rotation term that accounts for the interaction between chroma and hue differences, particularly in the blue region, improving perceptual accuracy.

Two readings were taken at the center of each specimen, and the mean values were used for analysis.

### Statistical analysis

Data were descriptively analyzed using measures of central tendency and dispersion (mean ± SD). Comparisons among resin composites were performed using the *F*-test (ANOVA) or the Kruskal–Wallis test, and comparisons between solutions were performed using the Mann–Whitney test. When a significant difference was detected by the *F*-test (ANOVA), multiple comparisons were conducted using Tukey’s test; when significance was detected by the Kruskal–Wallis test, multiple comparisons were performed using Conover’s test. The *F*-test (ANOVA) was selected when the numerical variable under analysis showed a normal distribution within each category and homogeneity of variances among resin composites, whereas the Kruskal–Wallis and Mann–Whitney tests were used due to the sample size of five specimens per composite–solution combination. Normality was assessed using the Shapiro–Wilk test, and homogeneity of variances using Levene’s *F*-test. The significance level was set at 5%. Data were entered into an Excel spreadsheet, and statistical analyses were performed using IBM SPSS (version 27) and MedCalc (version 20.104).

## Results

Table 2 presents the microhardness statistics according to the resin composite and the solutions used, as well as sorption and solubility.

**Table 2:** Microhardness analysis of the tested resin composites and sorption and solubility according to the evaluated resin composite.

Resin composite	Microhardness	Sorption	Solubility
		Mean ± SD	Mean ± SD
Beautifil II	65.89 ± 3.67^(^**^B^**^)^	13.36 ± 1.73^(^**^A^**^)^	1.13 ± 0.69
Beautifil Flow	41.73 ± 3.54 ^(^**^A^**^)^	17.78 ± 2.42 ^(^**^B^**^)^	2.03 ± 0.51
Z350XT	73.87 ± 2.07 ^(^**^C^**^)^	19.45 ± 3.08 ^(^**^B^**^)^	0.90 ± 1.30
Filtek Supreme	40.42 ± 1.83 ^(^**^A^**^)^	14.72 ± 0.89 ^(^**^A^**^)^	2.26 ± 1.44
p-value	p^(1)^ < 0.001*	p^(2)^ = 0.001*	p^(2)^ = 0.155

SD: standard deviation.

* Significant difference at the 5% level.

(1) *F*-test (ANOVA) with Tukey’s multiple comparisons test.

(2) Kruskal–Wallis test with Conover’s multiple comparisons test.

Note: Different letters in parentheses indicate statistically significant differences among the corresponding resin composites.

Microhardness was highest for the Z350XT resin composite, followed by the Beautifil II resin composite, and lowest for the Filtek Supreme and Beautifil Flow Plus resin composites (*p* < 0.001).

Sorption was significantly higher for the Z350XT resin composite and Beautifil Flow Plus resin composite than for the other two resin composites (*p* = 0.001). The mean solubility values for the four resin composites ranged from 0.90 to 2.26, with no significant differences among the resin composites (*p* > 0.05).

Tables 3 and 4 present the results for color change (ΔE) after 1 day (ΔE1) and 7 days (ΔE7) of immersion in the solutions. Table 3 shows that after 1 day of immersion, each resin composite exhibited greater color change in coffee. The significantly greatest color changes in coffee were observed for the high-viscosity resin composites (Z350XT and Beautifil II), whereas the low-viscosity resin composites (Filtek Supreme Flowable and Beautifil Flow Plus) showed lower color change (*p* = 0.001).

**Table 3:** Mean and standard deviation of ∆E and ∆E_00_ on day 1 according to the resin composite and the solution used.

Solution				
Color analysis	Resin composite	Water Mean ± SD	Coffee Mean ± SD	*p*-value
∆E	Beautifil II	0.21 ± 0.14^(^**^Aa^**^)^	10.17 ± 1.21 ^(^**^Bb^**^)^	*p*^(1)^ = 0.008*
	Beautifil Flow	0.56 ± 0.40 ^(^**^ABa^**^)^	4.98 ± 0.93 ^(^**^Ab^**^)^	*p*^(1)^ = 0.008*
	Z350XT	0.17 ± 0.08 ^(^**^Aa^**^)^	8.55 ± 0.93 ^(^**^Bb^**^)^	*p*^(1)^ = 0.008*
	Filtek Supreme	0.83 ± 0.52 ^(Ba)^	4.10 ± 0.72 ^(Ab)^	*p*^(1)^ = 0.008*
	p-value	p^(2)^ = 0.020*	p^(2)^ = 0.001*	
∆E_00_	Beautifil II	0.09 ± 0.08 ^(Aa)^	5.21 ± 0.65 ^(Bb)^	*p*^(1)^ = 0.008*
	Beautifil Flow	0.30 ± 0.20 ^(Aa)^	2.91 ± 0.48 ^(Ab)^	*p*^(1)^ = 0.008*
	Z350XT	0.09 ± 0.07 ^(Aa)^	4.70 ± 0.54 ^(Bb)^	*p*^(1)^ = 0.008*
	Filtek Supreme	0.31 ± 0.25 ^(Aa)^	2.45 ± 0.43 ^(Ab)^	*p*^(1)^ = 0.008*
	p-value	p^(2)^ = 0.070	p^(2)^ = 0.002*	

SD: standard deviation.

* Significant difference at the 5% level.

(1) Mann–Whitney test.

(2) Kruskal–Wallis test with Conover’s multiple comparisons test.

Note: Different superscript uppercase letters indicate statistically significant differences among the resin composites immersed in the same solution. Different superscript lowercase letters indicate statistically significant differences between the evaluated solutions (water and coffee).

**Table 4:** Mean and standard deviation of ∆E and ∆E_00_ on day 7 according to the resin composite and the solution used.

Solution				
Color analysis	Resin composite	Water Mean ± SD	Coffee Mean ± SD	*p*-value
∆E	Beautifil II	0.39 ± 0.21^(Aa)^	14.76 ± 1.59 ^(Bb)^	*p*^(1)^ = 0.008*
	Beautifil Flow	1.09 ± 0.57 ^(Ba)^	6.94 ± 1.00 ^(Ab)^	*p*^(1)^ = 0.008*
	Z350XT	0.38 ± 0.14 ^(Aa)^	13.43 ± 0.74 ^(Bb)^	*p*^(1)^ = 0.008*
	Filtek Supreme	1.24 ± 0.50 ^(Ba)^	5.54 ± 0.72 ^(Ab)^	*p*^(1)^ = 0.008*
	p-value	p^(2)^ = 0.003*	p^(2)^ = 0.001*	
∆E_00_	Beautifil II	0.20 ± 0.09 ^(Aa)^	8.16 ± 0.89 ^(Bb)^	*p*^(1)^ = 0.008*
	Beautifil Flow	0.50 ± 0.27 ^(Ba)^	4.57 ± 0.42 ^(Ab)^	*p*^(1)^ = 0.008*
	Z350XT	0.28 ± 0.06 ^(Aa)^	7.31 ± 0.44 ^(Bb)^	*p*^(1)^ = 0.008*
	Filtek Supreme	0.54 ± 0.24 ^(Ba)^	3.29 ± 0.69 ^(Ab)^	*p*^(1)^ = 0.008*
	p-value	p^(2)^ = 0.007	p^(2)^ = 0.001*	

SD: standard deviation.

* Significant difference at the 5% level.

(1) Mann–Whitney test.

(2) Kruskal–Wallis test with Conover’s multiple comparisons test.

Note: Different superscript uppercase letters indicate statistically significant differences among the resin composites immersed in the same solution. Different superscript lowercase letters indicate statistically significant differences between the evaluated solutions (water and coffee).

Table 4 presents the mean ΔE values after 7 days of immersion (ΔE7). Similar to the 1-day immersion analysis, all resin composites exhibited greater color change when immersed in coffee. The high-viscosity resin composites (Z350XT and Beautifil II) exhibited greater pigmentation than their low-viscosity counterparts (*p* = 0.001). No statistically significant differences were observed among the tested low-viscosity resin composites.

## Discussion

The first null hypothesis of the study, which proposed no difference in microhardness among the resin composites, was rejected. The conventional high-viscosity resin composite (Filtek Z350 XT) showed a significantly higher microhardness than the “bioactive” (Beautifil II) and low-viscosity resin composites (Filtek Supreme Flowable and Beautifil Flow Plus). Microhardness measurements, such as those obtained by the Vickers test (VHN), are considered a critical indicator of mechanical properties, given their positive correlation with wear resistance and, consequently, with the clinical longevity of restorations [21]. Resin composites with lower hardness show greater susceptibility to surface defects and fractures under masticatory forces, underscoring the relevance of this finding [21]. From a clinical perspective, higher hardness values are generally associated with greater resistance to occlusal wear, particularly in posterior restorations, where functional load is greater [22].

The higher hardness of the conventional resin composite (Filtek Z350 XT) compared with the BAG-filled resin composites (Beautifil II and Beautifil Flow Plus) is consistent with the current literature [23]. This trade-off between bioactivity and mechanical performance has been widely discussed, as the incorporation of reactive fillers may disrupt the integrity of the polymer network or reduce filler–matrix bonding efficiency [24]. The main mechanism underlying bioactivity involves incorporating particles such as bioactive glass or ion-releasing resin composites, which may increase reactivity and result in inferior mechanical properties [23]. In parallel, the marked decrease in microhardness observed in the low-viscosity versions reflects the reduced inorganic filler content in their formulations (Table 4). This statistical difference between high- and low-viscosity resin composites is primarily determined by this feature, as higher filler loading increases hardness, whereas reducing filler content is essential to obtain low viscosity [25].

These findings are in line with previous studies emphasizing that increased filler content improves physicomechanical properties [17, 18]. However, not only the amount of filler, but also its size and aggregation state determine the superior microhardness performance observed for Filtek Z350 XT. Nanofilled systems, such as Filtek Z350 XT, present improved filler packing and dispersion, which enhances mechanical properties by promoting a more homogeneous stress distribution and reducing crack propagation [26].

In addition, microhardness serves as a reliable indirect method to assess polymerization efficiency. Maintaining fundamental physical characteristics, such as crosslink density and degree of conversion (DC), is crucial for restoration durability, and a positive correlation between DC and surface hardness has been reported [27]. The values observed in the present study are comparable to those reported in previous investigations evaluating conventional nanofilled composites, reinforcing the reliability of the findings [20]. Hardness measurements obtained by microindentation testing support that increased crosslinking among monomers is a monitorable and essential factor for mechanical strength, justifying the selection of higher-hardness resin composites for areas subjected to occlusal stress [18].

The second null hypothesis was also rejected, as statistically significant differences were observed in the sorption analysis of the high-viscosity resin composites. Thus, Z350XT and Beautifil Flow Plus exhibited higher sorption than Beautifil II and Filtek Supreme Flowable. Water uptake acts as a plasticizer and may also cause material expansion and hydrolysis of components such as silane. These changes may lead to dimensional alterations, marginal failures, and resin composite discoloration [28]. Clinically, excessive water sorption is undesirable, as it accelerates degradation mechanisms and compromises restoration integrity over time [16].

In the present study, the conventional high-viscosity resin composite Z350XT showed higher sorption than its low-viscosity version (FS). This pattern differs from the most frequent findings in the literature, as low-viscosity resin composites typically have lower filler content and a higher proportion of hydrophilic diluent monomers (such as TEGDMA), which would be expected to increase water uptake [29]. Nevertheless, a study assessing Filtek Z350 XT (conventional) reported higher sorption and solubility values than more modern bulk-fill composites (such as Filtek Bulk Fill or Filtek One Bulk Fill Restorative) [30]. Furthermore, although a statistically significant difference was observed between the resin composites, the magnitude of this variation was relatively small (below 4.73 µg/mm³), especially when compared to the standard deviation of the Z350XT resin composite (3.08 µg/mm³). In addition, all resin composites presented water sorption values well below the maximum limit established by ISO 4049 (40 µg/mm³), indicating that the results are within clinically acceptable thresholds. Beautifil Flow Plus presented the second-highest sorption value. This result is expected for ion-releasing resin composites, as their matrices are often more hydrophilic to allow ionic exchange and surface reactions of S-PRG glass particles, which in turn increases water sorption [30, 31].

The third null hypothesis was not rejected, as no statistically significant differences were observed among the tested groups (*p* > 0.05). Solubility is determined by monomer structure, fillers, and degree of conversion [32]. Even in resin composites with high sorption, the polymer network may be sufficiently crosslinked (high degree of conversion) to minimize the release of soluble components [28]. The compounds of the evaluated BAG-filled resin composites could be associated with changes in resin composite solubility. The literature has reported that fluoride-releasing composites may show higher solubility because water diffusion is required for effectiveness [33]. However, this was not observed in the present study. According to ISO 4049:2019, the maximum acceptable solubility is 7.5 µg/mm³. Therefore, all resin composites analyzed in this study complied with the parameters established by this standard. This finding suggests that incorporating BAG into the evaluated resin composites did not compromise their chemical stability within acceptable limits.

The fourth null hypothesis was also rejected, as statistically significant differences were found in the color stability of the tested resin composites when immersed in coffee. Among the evaluated groups, the most pronounced color changes after 7 days of immersion occurred for Beautifil II and Z350XT resin composites, regardless of the color analysis method used (ΔE or ΔE00). These findings are consistent with the literature, as coffee is cited as one of the main agents responsible for extrinsic staining of high-viscosity resin composites [34]. In addition, the color change increased progressively over time. These results indicate that exposure time is a crucial factor, allowing greater diffusion of low-molecular-weight pigments contained in the beverage [35]. Accordingly, these aspects should be considered when selecting resin composites that exhibit smaller changes over time when exposed to different staining agents.

From a clinical perspective, color change should be interpreted based on perceptibility and acceptability thresholds. For CIELab measurements, ΔE values greater than approximately 1.2 are considered perceptible, while values above 2.7 are considered clinically unacceptable. Whereas for ΔE₀₀, color differences of approximately 0.8 are considered perceptible, while values above 1.8 are regarded as unacceptable [36]. In the present study, all groups immersed in coffee exceeded these thresholds, indicating that the observed discoloration would be noticeable and clinically unacceptable over time. This finding reinforces the strong staining potential of coffee and highlights the importance of resin composite selection in esthetic areas.

However, an important aspect to consider is that, in clinical trials, a restoration is only deemed clinically unacceptable and in need of replacement when the color change is severe. In the present study, however, the samples were subjected to prolonged immersion in coffee, a condition that does not directly reflect the actual exposure time of this beverage in the oral cavity. Therefore, the results should be interpreted as a comparative analysis aimed at assessing whether the staining of resin composites modified with BAG-filled particles exhibits behavior similar to that of resin composites known for their good clinical longevity.

The current study found no statistically significant differences between the BAG-filled resin composites and the conventional resin composite, in both their high- and low-viscosity versions. Clinical studies evaluating the Z350XT resin composite, with follow-up periods of up to 6 years, have demonstrated high survival rates of restorations, including for the color match criterion [37, 38].

Limitations of an *in vitro* study include the absence of real clinical conditions that may directly influence the behavior of the evaluated resin composites, such as variations in salivary pH, the presence of biofilm, and masticatory loading. In addition, the short evaluation period may have affected the results, limiting the interpretation of resin composite performance in the medium and long term. Future studies should include long-term aging protocols, mechanical cycling, and biofilm interaction to better simulate clinical conditions and further validate these findings.

## Conclusion

BAG-filled resin composites exhibit properties comparable to those of conventional resin composites. The Z350XT resin composite showed higher microhardness; however, for the other evaluated parameters (sorption, solubility, and color stability in coffee), the tested “bioactive” resin composites exhibited values within the limits recommended by ISO 4049:2019 and were comparable to those of the reference conventional high or low-viscosity restorative resin composite.

## Data Availability

The data are available upon request.
